# Health policy implications of corporate social responsibility provisions in international investment agreements

**DOI:** 10.2471/BLT.23.290419

**Published:** 2023-12-08

**Authors:** Takwa Tissaoui, Teresa Davis, Helen Trevena, Anne Marie Thow

**Affiliations:** a Menzies Centre for Health Policy and Economics, Sydney School of Public Health, The University of Sydney, Camperdown 2050, New South Wales, Australia.; b Discipline of Marketing, University of Sydney Business School, Sydney, Australia.

## Abstract

**Objective:**

To analyse and classify inclusions of corporate social responsibility in international investment agreements, especially inclusions with reference to public health.

**Method:**

We extracted the text of international investment agreements containing corporate social responsibility inclusions from the electronic database of investment treaties. We conducted a documentary analysis of the corporate social responsibility inclusions, and we developed a typology categorizing inclusions based on level of detail and reference to international commitments.

**Findings:**

Of the 3816 agreements signed as of October 2023, 127 agreements contain corporate social responsibility inclusions. Since the first inclusion of corporate social responsibility in 2008, the percentage of agreements containing such inclusion signed each year has steadily increased from 4.6% (4/86) in 2008 to 42.8% (21/49) in 2018 and 33.3% (3/9) in 2023. Using the typology we developed, we categorized the level of detail as follows: nine were minimal, 27 were low, 35 were low-medium, 107 were medium, 11 were medium-high and seven were high. Health is mentioned in 36 of these inclusions.

**Conclusion:**

This analysis indicates that international investment agreements increasingly incorporate a high level of detail on expectations regarding investors’ corporate social responsibility. Such provisions offer a potential tool to increase government guidance and accountability of global corporations, including with respect to governments’ public health objectives.

## Résumé

**Objectif:**

Analyser et classer les dispositions relatives à la responsabilité sociale des entreprises dans les accords internationaux d’investissement, en particulier celles relatives à la santé publique.

**Méthodes:**

Nous avons extrait de la Base de données électronique des traités d’investissement (EDIT) le texte d’accords internationaux d’investissement contenant des dispositions relatives à la responsabilité sociale des entreprises. Nous avons effectué une analyse documentaire des dispositions relatives à la responsabilité sociale des entreprises et avons élaboré une typologie classant ces dispositions selon leur niveau de détail et leur référence à des engagements internationaux.

**Résultats:**

Sur les 3816 accords signés en date d’octobre 2023, 127 contiennent des dispositions relatives à la responsabilité sociale des entreprises. Depuis l’inclusion de la première disposition de responsabilité sociale des entreprises en 2008, le pourcentage d’accords signés chaque année et contenant ce type de disposition a augmenté régulièrement, passant de 4,6 % (4/86) en 2008 à 42,8% (21/49) en 2018 et 33,3% (3/9) en 2023. À l’aide de la typologie que nous avons élaborée, nous avons classé le niveau de détail comme suit : neuf niveaux de détail étaient minimes, 27 étaient faibles, 35 étaient faibles à moyens, 107 étaient moyens, 11 étaient moyens à élevés et sept étaient élevés. La santé est mentionnée dans 36 de ces dispositions.

**Conclusion:**

Cette analyse indique que les accords internationaux d’investissement intègrent de plus en plus souvent un niveau élevé de détails sur les attentes en matière de responsabilité sociale des entreprises. Ces dispositions constituent un outil potentiel pour renforcer les orientations gouvernementales et la responsabilité des entreprises internationales, notamment en ce qui concerne les objectifs de santé publique des gouvernements.

## Resumen

**Objetivo:**

Analizar y clasificar las inclusiones de la responsabilidad social empresarial en los acuerdos internacionales de inversión, en especial las inclusiones con referencia a la salud pública.

**Método:**

Se extrajo de la Base de Datos Electrónica de Tratados de Inversión el texto de los acuerdos internacionales de inversión que incluían la responsabilidad social empresarial. Se realizó un análisis documental de las inclusiones de la responsabilidad social empresarial y se elaboró una tipología que clasifica las inclusiones en función del nivel de detalle y la referencia a los compromisos internacionales.

**Resultados:**

De los 3816 acuerdos firmados hasta octubre de 2023, 127 acuerdos contienen inclusiones de responsabilidad social empresarial. Desde la primera inclusión de la responsabilidad social empresarial en 2008, el porcentaje de acuerdos firmados cada año que contienen esta inclusión ha aumentado de manera constante, pasando del 4,6% (4/86) en 2008 al 42,8% (21/49) en 2018 y al 33,3% (3/9) en 2023. Mediante la tipología que se desarrolló, se categorizó el nivel de detalle de la siguiente manera: nueve fueron mínimos, 27 fueron bajos, 35 fueron bajos-medios, 107 fueron medios, 11 fueron medios-altos y siete fueron altos. La salud se menciona en 36 de estas inclusiones.

**Conclusión:**

Este análisis indica que los acuerdos internacionales de inversión incorporan cada vez más un alto nivel de detalle sobre las expectativas en materia de responsabilidad social empresarial de los inversores. Estas disposiciones ofrecen una herramienta potencial para aumentar la orientación de los gobiernos y la rendición de cuentas de las empresas mundiales, incluso con respecto a los objetivos de salud pública de los gobiernos.

## ملخص

**الغرض:**

تحليل وتصنيف حالات تضمين المسؤولية الاجتماعية للشركات في اتفاقيات الاستثمار الدولية، وخاصة التي تتعلق بالصحة العامة.

**الطريقة:**

قمنا باستخراج نصوص اتفاقيات الاستثمار الدولية، التي تحتوي على شروط تضمين المسؤولية الاجتماعية للشركات، من قاعدة البيانات الإلكترونية لمعاهدات الاستثمار. وأجرينا تحليلاً وثائقيًا لحالات تضمين المسؤولية الاجتماعية للشركات، وقمنا بتطوير نمط لتصنيف حالات التضمين بناءً على مستوى التفاصيل، والإشارة إلى الالتزامات الدولية.

**النتائج:**

من بين 3816 اتفاقية تم التوقيع عليها منذ أكتوبر/تشرين أول 2023، هناك 127 اتفاقية تحتوي على حالات تضمين للمسؤولية الاجتماعية للشركات. منذ أول تضمين للمسؤولية الاجتماعية للشركات في عام 2008، فإن نسبة الاتفاقيات الموقعة كل عام التي تحتوي على هذا التضمين، زادت بشكل مطرد من %4.6 (4/86) في عام 2008، إلى %42.8 (21/49) في عام 2018، و%33.3 (3/ 9) في عام 2023. وباستخدام النمط الذي طورناه، قمنا بتصنيف مستوى التفاصيل كما يلي: 9 بسيطة، و27 منخفضة، و35 منخفضة إلى متوسطة، و107 متوسطة، و11 متوسطة إلى مرتفعة، و7 مرتفعة. وتمت الإشارة إلى الصحة في 36 من حالات التضمين تلك.

**الاستنتاج:**

يشير هذا التحليل إلى أن اتفاقيات الاستثمار الدولية تتضمن بشكل متزايد مستوى عالٍ من التفاصيل حول التوقعات المتعلقة بالمسؤولية الاجتماعية لشركات المستثمرين. توفر مثل هذه الفقرات أداة محتملة لزيادة التوجيه الحكومي، ومساءلة الشركات العالمية، بما في ذلك ما يتعلق بأهداف الصحة العامة للحكومات.

## 摘要

**目的:**

分析国际投资协议中企业社会责任的内容并对其进行分类,特别是与公共卫生相关的内容**。**

**方法:**

我们从投资条约电子数据库中摘录了包含企业社会责任内容的国际投资协议的文本。我们对企业社会责任内容进行了文献分析,并根据详细程度和对国际承诺的参考,开发了一种类型分类法。

**结果:**

在截至 2023 年 10 月签署的 3816 份协议中,有 127 份协议包含企业社会责任内容。自 2008 年首次纳入企业社会责任以来,每年签署的包含企业社会责任的协议的比例稳步上升,从 2008 年的 4.6% (4/86) 上升到 2018 年的 42.8% (21/49) 和 2023 年的 33.3% (3/9)。使用我们开发的类型分类法,我们对详细程度进行了分类,如下:9 个为最低,27 个为较低,35 个为中低,107 个为中等,11 个为中高,7 个为较高。其中 36 项协议提到了健康。

**结论:**

这一分析表明,国际投资协议越来越多地纳入了对投资者的企业社会责任的期望的高度详细的内容。这些条款提供了潜在的工具,可以加强政府对国际公司的指导和问责,包括关于政府的公共卫生目标方面。

## Резюме

**Цель:**

Проанализировать и классифицировать включения корпоративной социальной ответственности в международные инвестиционные договоры, в частности включения в отношении общественного здравоохранения.

**Методы:**

Из электронной базы данных инвестиционных соглашений были извлечены тексты международных инвестиционных договоров, содержащих положения о корпоративной социальной ответственности. Проведен документальный анализ включений, касающихся корпоративной социальной ответственности, и разработана типология, классифицирующая включения по степени детализации и ссылкам на международные обязательства.

**Результаты:**

Из 3816 соглашений, подписанных по состоянию на октябрь 2023 года, 127 соглашений содержат положения о корпоративной социальной ответственности. С момента первого включения корпоративной социальной ответственности в 2008 году доля подписываемых ежегодно соглашений с таким включением неуклонно росла: с 4,6% (4/86) в 2008 году до 42,8% (21/49) в 2018 году и 33,3% (3/9) в 2023 году. На основе разработанной типологии уровень детализации был распределен следующим образом: девять – минимальный, 27 – низкий, 35 – низкий-средний, 107 – средний, 11 – средний-высокий и семь – высокий. Информация о здоровье упоминается в 36 из этих включений.

**Вывод:**

Результаты проведенного анализа свидетельствуют о том, что в международных инвестиционных договорах все чаще встречается высокая степень детализации ожиданий в отношении корпоративной социальной ответственности инвесторов. Такие положения являются возможным инструментом для усиления государственного руководства и подотчетности глобальных корпораций, в том числе в отношении целей правительства в области общественного здравоохранения.

## Introduction

The term corporate social responsibility reflects a shift in the norms and expectations of the public and governments regarding corporate behaviour and its impact on objectives including public health. Globalization, along with increasing public awareness of the potential negative impacts of corporate investors, particularly for the environment and public health, have facilitated this shift.^1^^,^^2^ Beyond their responsibility to shareholders, corporations are now expected to integrate goals beyond profit-making into their operations.^3^ One channel for companies seeking to achieve these goals is through corporate social responsibility, which broadly spans corporate citizenship, cause-related marketing, stakeholder management, universal rights and sustainable development.^4^ In this paper, we use the European Commission’s understanding of corporate social responsibility, defined as the responsibility of enterprises for their impacts on society.^5^ Guidelines, standards and principles of corporate social responsibility have played a key role in defining and legitimizing the expected obligations of businesses at the international and national levels.^6^ Soft law instruments such as the United Nations (UN) Global Compact^7^ have formalized the promotion of corporate social responsibility,^2^ while governments have also contributed through taking a more holistic approach to development in line with the sustainable development goals (SDGs) including those relating to public health.^8^ The Organisation for Economic Co-operation and Development (OECD), has particularly played a key role in supporting governments in the development of international investment agreement provisions that place a responsibility on the investor. The OECD introduced the term responsible business conduct as an alternative to corporate social responsibility, in cooperation with nongovernmental organizations, businesses and trade unions.^9^ Responsible business conduct refers to “making a positive contribution to economic, environmental and social progress with a view to achieving sustainable development and avoiding and addressing adverse impacts related to an enterprise’s direct and indirect operations, products or services.”^5^

Recently, heightened government interest in corporate social responsibility – notably for its potential to support the achievement of goals relevant to sustainable development and public health – has led to new provisions within international investment agreements. These provisions place expectations on corporate investors regarding their corporate social responsibility activities, including with respect to public health considerations. International investment agreements are intergovernmental treaties that promote and remove barriers to foreign direct investment and trade, and include bilateral investment treaties, and free trade and investment agreements.^10^ The focus of international investment agreements is the protection of investor rights as a means to promote and attract foreign investment, and many include a mechanism for investor–State dispute settlement, through which investors can submit disputes against States.^11^ These provisions have been used to challenge public health policies affecting investors, leading to widespread concern regarding negative implications of international investment agreements for public health.^11^^–^^13^ For example, Phillip Morris International disputed tobacco legislation on plain packaging in Australia and Uruguay, costing these governments millions of dollars in defending their legitimate public health policy measures.^14^ Therefore, international investment agreements are considered to be a commercial determinant of health.^15^

A key issue underpinning public health concerns about international investment agreements is their nearly exclusive focus on protecting investor rights. The inclusion of corporate social responsibility provisions in international investment agreements represent efforts to balance corporate investor rights with government priorities, through the creation of investor obligations that encourage investors to align with government policy objectives, including public health.^8^^,^^16^^,^^17^ Furthermore, these provisions in international investment agreements are an avenue to transform soft law encouragement of corporate social responsibility to a form of hard law, to ensure that corporate social responsibility aligns with government priorities.^2^ This movement has been further enabled by the entrenchment of public health in sustainable development objectives.^18^ Corporate social responsibility has the potential to contribute to population health and health equity through societal and multisectoral approaches.^19^ However, concerns remain about the misalignment between corporate motivations and public health objectives, particularly the tendency towards a disengaged corporate social responsibility weakly linked to corporate operations, rather than an engaged corporate social responsibility^20^ that has a direct impact on operations.^21^^–^^25^ Evidence shows that corporate social responsibility initiatives such as self-regulatory pledges do not generate substantial improvements in public health.^26^ At the same time, and in direct contrast to stated aims of corporate social responsibility to improve health, evidence suggests the influence of multinational corporations in watering down national health policy measures.^27^ The resulting disconnect between corporate social responsibility activities and government policy objectives for improving public health has contributed to increasing attention to the potential for governments to play a more active role in monitoring corporate social responsibility, including through international investment agreements.

As a result, governments’ efforts to increase companies’ obligations and accountability in respect to corporate social responsibility through international investment agreements have implications for public health. Understanding these new inclusions and the extent to which they incorporate health can contribute to identifying avenues for improving corporate social responsibility from a health perspective. Here we examine the nature of these inclusions, and potential implications for public health.

## Methods

To identify corporate social responsibility in international investment agreements, we searched the Electronic Database of Investment Treaties^28^ in October 2023 for agreements containing mention of “corporate social responsibility,” “social responsibility” and/or “responsible business conduct”. Agreements were included if they were signed or in force and contained one of the above-mentioned search terms. Agreements were excluded if they were a model agreement or if the search term was found only in the annex. We imported the text of the eligible agreements into NVivo (Lumivero, Denver, United States of America) and coded text specific to these three terms, along with the parties involved, the location of the terms within the agreement, and mentions of health and sustainable development within the agreements. We also compiled the agreements into a data set that detailed the parties to the agreements; dates the agreements were signed; type of agreement; and the World Health Organization (WHO) regions and World Bank gross national income categorizations of those parties.

To identify the existing research on corporate social responsibility inclusions in international investment agreements, we reviewed the academic literature between June and October 2022 (searching Google Scholar, the ProQuest Central database and the legal database HeinOnline) and grey literature (searching Google) using the search terms “corporate social responsibility” and “international investment agreements” (including similar terms such as “treaty”). We deemed an article or report to be relevant if it examined the relationship between corporate social responsibility and public health, or the inclusion of corporate social responsibility provisions in international investment agreements. One author extracted relevant information to the referencing software Zotero (Corporation for Digital Scholarship, Vienna, United States) and prepared initial review reports of the key considerations relevant to, and the historical development of, corporate social responsibility inclusions in international investment agreements.

Informed by the review of the agreements, the literature review and the policy options detailed by the United Nations Conference on Trade and Development in 2015,^29^ we identified two important features of corporate social responsibility inclusions: (i) reference to guidelines, standards and principles of corporate social responsibility; and (ii) a description of corporate activities and obligations States seek to place on corporate investors. We then conducted a documentary analysis of the corporate social responsibility inclusions in the extracted international investment agreements. First, one author coded the data to these two characteristics, as well as other descriptive features including parties to the agreement, agreement type, year signed and placement of inclusion (in the preamble or as a provision outside of it), using NVivo. We analysed the extent to which the two features were demonstrated in the text of the inclusions, and created typology categories that reflected the spectrum of the features evident in the data (Table 1). We reviewed and discussed these typology categories, with reference to the literature. One author then recoded the data to these detailed typology categories, and analysed the data to examine the temporal evolution of the corporate social responsibility inclusions and leading parties against the different types.

**Table 1:** Typology of corporate social responsibility inclusions in international investment agreements, 2015–2021

Level of detail	Typology criteria	No. of inclusions	Example of corporate social responsibility inclusion
Minimal	No reference to external guidelines, standards or principles; and no description of corporate social responsibility activities	9	China–EU Comprehensive Agreement on Investment, 2021 Preambulatory Clause COMMITTED to encourage enterprises to respect corporate social responsibility or responsible business conduct.
Low	Reference to external guidelines, standards or principles but unnamed; and no description of corporate social responsibility activities	27	Australia–Indonesia Comprehensive Economic Partnership Agreement, 2019 Article 14.17. Corporate Social Responsibility Each Party reaffirms the importance of encouraging enterprises operating within its territory or subject to its jurisdiction to voluntarily incorporate into their internal policies those internationally recognized standards, guidelines and principles of corporate social responsibility that have been endorsed or are supported by that Party.
Low-Medium	Reference to named external guidelines, standards or principles with no description of corporate social responsibility; or includes brief description of corporate social responsibility but no reference to guidelines, standards or principles	35	Agreement for the Reciprocal Promotion and Protection of Investments Between the Argentine Republic and the United Arab Emirates, 2018 Article 17. Corporate Social Responsibility The Parties, being mindful of internationally recognized corporate social responsibility standards, guidelines and principles, including the Organisation for Economic Cooperation and Development (OECD) Guidelines for Multinational Enterprises, shall endeavour to encourage enterprises doing business in its territory or subject to its jurisdiction to voluntarily include said standards, guidelines and principles.
Medium	Reference to named or unnamed external guidelines, standards or principles; and brief description of corporate social responsibility activities	107	Agreement for the Promotion and Reciprocal Protection of Investments Between Canada and the Republic of Guinea, 2015 Article 16. Corporate Social Responsibility Each Party should encourage enterprises operating within its territory or subject to its jurisdiction to voluntarily incorporate internationally recognized standards of corporate social responsibility in their practices and internal policies, such as statements of principle that have been endorsed or are supported by the Parties. These principles address issues such as labour, the environment, human rights, community relations and anti-corruption. Free Trade Agreement Between the European Union and the Republic of Singapore, 2018 Article 12.11. Trade and Investment Promoting Sustainable Development 4. When promoting trade and investment, the Parties should make special efforts to promote corporate social responsibility practices which are adopted on a voluntary basis. In this regard, each Party shall refer to relevant internationally accepted principles, standards or guidelines to which it has agreed or acceded, such as the Organization for Economic Cooperation and Development Guidelines for Multinational Enterprises, the UN Global Compact, and the ILO Tripartite Declaration of Principles concerning Multinational Enterprises and Social Policy. The Parties commit to exchanging information and cooperating on promoting corporate social responsibility.
Medium-High	No reference to external guidelines, standards or principles; and comprehensive description of corporate social responsibility	11	Agreement on Cooperation and Facilitation of Investment Between the Federative Republic of Brazil and the United Mexican States, 2015 Article 12. Corporate Social Responsibility 12.1 Investors and their investments shall strive to achieve the highest possible level of contribution to the sustainable development of the Host State and the local community, through the adoption of a high degree of socially responsible practices, based on the voluntary principles and standards set out in this Article and internal policies, such as statements of principle that have been endorsed or are supported by the Parties. 12.2 The investors and their investments shall endeavour to comply with the following voluntary principles and standards for a responsible business conduct and consistent with the laws adopted by the Host State: a) Contribute to the economic, social and environmental progress, aiming at achieving sustainable development; b) Respect the internationally recognized human rights of those involved in the companies’ activities; c) Encourage local capacity-building through close cooperation with the local community; d) Encourage the creation of human capital, especially by creating employment opportunities and offering professional training to workers; e) Refrain from seeking or accepting exemptions that are not established in the legal or regulatory framework relating to human rights, environment, health, security, work, tax system, financial incentives, or other issues; f) Support and advocate for good corporate governance principles, and develop and apply good corporate governance practices, including anti-corruption measures; g) Develop and implement effective self-regulatory practices and management systems that foster a relationship of mutual trust between the companies and the societies in which their operations are conducted; h) Promote the knowledge of and the adherence, by workers, to the corporate policy, through appropriate dissemination of this policy, including professional training program[me]s; i) Refrain from discriminatory or disciplinary action against employees who submit grave reports to the board or, whenever appropriate, to the competent public authorities, about practices that violate the law or corporate policy; j) Encourage, whenever possible, business associates, including service providers and outsources, to apply the principles of business conduct consistent with the principles provided for in this Article; and k) Refrain from any undue interference in local political activities.
High	With reference to named or unnamed external recognized guidelines, principles; and comprehensive description of corporate social responsibility	7	Brazil–Ethiopia Agreement on Cooperation and Facilitation of Investments, 2018 Section II. Regulatory Measures and Risk Mitigation Article 14. Corporate Social Responsibility 1. Investors and their investment shall strive to achieve the highest possible level of contribution to the sustainable development of the Host State and the local community, through the adoption of a high degree of socially responsible practices, based on the principles and standards set out in this Article and the OECD Guidelines for Multinational Enterprises (MNEs) as may be applicable on the State Parties. 2. Investors and their investment shall endeavour to comply with the following principles and standards for a responsible business conduct and consistent with the laws adopted by the Host State: a) Contribute to the economic, social and environmental progress, aiming at achieving sustainable development; b) Respect the internationally recognized human rights of those involved in the investors' activities; c) Encourage local capacity building through close cooperation with the local community; d) Encourage the creation of human capital, especially by creating employment opportunities and offering professional training to workers; e) Refrain from seeking or accepting exemptions that are not established in the legal or regulatory framework relating to human rights, environment, health, security, work, tax system, financial incentives, or other issues; f) Support and advocate for good corporate governance principles, and develop and apply good practices of corporate governance; g) Develop and implement effective self-regulatory practices and management systems that foster a relationship of mutual trust between the investment and the societies in which its operations are conducted; h) Promote the knowledge of and the adherence to, by workers, the corporate policy, through appropriate dissemination of this policy, including programs for professional training; i) Refrain from discriminatory or disciplinary action against employees who submit grave reports to the board or, whenever appropriate, to the competent public authorities, about practices that violate the law or corporate policy; j) Encourage, whenever possible, business associates, including service providers and outsources, to apply the principles of business conduct consistent with the principles provided for in this Article; and k) Refrain from any undue interference in local political activities.

EU: European Union; ILO: International Labour Organization; OECD: Organisation for Economic Co-operation and Development; UN: United Nations.

## Results

Corporate social responsibility provisions in international investment agreements are relatively new, but have increased rapidly in numbers. Of the 3816 international investment agreements signed as of October 2023 (the first agreement dating back to 1948),^28^ 127 agreements (Box 1; available at: https://www.who.int/publications/journals/bulletin/) contain corporate social responsibility inclusions. The first example of a corporate social responsibility provision appeared in 2005 in the International Institute for Sustainable Development Model International Agreement on Investment for Sustainable Development.^30^ The first agreements to contain corporate social responsibility and responsible business conduct inclusions were the Free Trade Agreement between Canada and the Republic of Colombia (2008) and the Free Trade Agreement between the European Free Trade Association States and the Republic of Serbia (2009), respectively.

Box 1 **Identified international investment agreements that included corporate social responsibility engagements 2008–2023**

***2008***

Canada – Colombia Free Trade Agreement

Canada – Peru Free Trade Agreement

Caribbean Forum – European Community Economic Partnership Agreement

Economic Community of West African States Supplementary Act on Investments

***2009***

Albania – European Free Trade Association Free Trade Agreement

European Free Trade Association – Serbia Free Trade Agreement

***2010***

Canada – Panama Free Trade Agreement

European Union – Republic of Korea Free Trade Agreement

***2011***

European Free Trade Association – Hong Kong Special Administrative Region, China, Free Trade Agreement

European Free Trade Association – Montenegro Free Trade Agreement

Kosovo – Switzerland Bilateral Investment Treaty

***2012***

Central America – European Union Association Agreement

Colombia – Ecuador – European Union – Peru Free Trade Agreement

***2013***

Benin – Canada Bilateral Investment Treaty

Bosnia and Herzegovina – European Free Trade Association Free Trade Agreement

Canada – Honduras Free Trade Agreement

China – Switzerland Free Trade Agreement

Colombia – Costa Rica Free Trade Agreement

Colombia – Panama Free Trade Agreement

European Free Trade Association – Costa Rica – Panama Free Trade Agreement

Netherlands – United Arab Emirates Bilateral Investment Treaty

***2014***

Cameroon – Canada Bilateral Investment Treaty

Canada – Côte d'Ivoire Bilateral Investment Treaty

Canada – Mali Bilateral Investment Treaty

Canada – Nigeria Bilateral Investment Treaty

Canada – Republic of Korea Free Trade Agreement

Canada – Senegal Bilateral Investment Treaty

Canada – Serbia Bilateral Investment Treaty

Colombia – France Bilateral Investment Treaty

European Union – Georgia Association Agreement

European Union – Republic of Moldova Association Agreement

European Union – Ukraine Association Agreement

Georgia – Switzerland Bilateral Investment Treaty

Pacific Alliance Additional Protocol

***2015***

Angola – Brazil Agreement on Cooperation and Facilitation of Investments

Brazil – Chile Agreement on Cooperation and Facilitation of Investments

Brazil –Colombia Agreement on Cooperation and Facilitation of Investments

Brazil – Malawi Agreement on Cooperation and Facilitation of Investments

Brazil – Mexico Agreement on Cooperation and Facilitation of Investments

Brazil – Mozambique Agreement on Cooperation and Facilitation of Investments

Burkina Faso – Canada Bilateral Investment Treaty

Canada – Guinea Bilateral Investment Treaty

European Union – Kazakhstan Enhanced Partnership and Cooperation Agreement

Kosovo – North Macedonia Bilateral Investment Treaty

***2016***

Albania – Kosovo Bilateral Investment Treaty

Argentina – Qatar Bilateral Investment Treaty

Brazil – Peru Economic and Trade Expansion Agreement

Canada – European Union Comprehensive Economic and Trade Agreement

Canada – Mongolia Bilateral Investment Treaty

Chile – Uruguay Free Trade Agreement

European Free Trade Association – Georgia Free Trade Agreement

European Free Trade Association – Philippines Free Trade Agreement

European Union – South African Development Community Economic Partnership Agreement

Morocco – Nigeria Bilateral Investment Treaty

Nigeria – Singapore Bilateral Investment Treaty

Trans-Pacific Partnership (TPP)

**2017**

Argentina – Chile Free Trade Agreement

Armenia – European Union Comprehensive and Enhanced Partnership Agreement

Canada – Chile Free Trade Agreement

Chile – Hong Kong Special Administrative Region Investment Agreement

Costa Rica – United Arab Emirates Bilateral Investment Treaty

Czechia – Islamic Republic of Iran Bilateral Investment Treaty

Hungary – Islamic Republic of Iran Bilateral Investment Treaty

Intra-MERCOSUR Investment Facilitation Protocol

Pacific Agreement on Closer Economic Relations (PACER) Plus

***2018***

Argentina – Japan Bilateral Investment Treaty

Argentina – United Arab Emirates Bilateral Investment Treaty

Australia – Peru Free Trade Agreement

Belarus – India Bilateral Investment Treaty

Brazil – Chile Free Trade Agreement

Brazil – Ethiopia Agreement on Cooperation and Facilitation of Investments

Brazil – Guyana Agreement on Cooperation and Facilitation of Investments

Brazil – Suriname Agreement on Cooperation and Facilitation of Investments

Canada – Kosovo Bilateral Investment Treaty

Comprehensive and Progressive Agreement for Trans-Pacific Partnership

Economic Community of West African States Common Investment Code

Ecuador – European Free Trade Association Free Trade Agreement

European Free Trade Association – Indonesia Free Trade Agreement

European Union – Japan Economic Partnership

European Union – Singapore Free Trade Agreement

India – China, Taiwan Bilateral Investment Treaty

Indonesia – Singapore Bilateral Investment Treaty

Lithuania – Türkiye Bilateral Investment Treaty

Serbia – Türkiye Bilateral Investment Treaty

Singapore – Sri Lanka Free Trade Agreement

United States – Mexico – Canada Agreement

***2019***

Australia – Hong Kong Special Administrative Region, China, Bilateral Investment Treaty

Australia – Indonesia Comprehensive Economic Partnership Agreement

Belarus – Hungary Bilateral Investment Treaty

Brazil – Ecuador Agreement on Cooperation and Facilitation of Investments

Brazil – Morocco Agreement on Cooperation and Facilitation of Investments

Brazil – United Arab Emirates Agreement on Cooperation and Facilitation of Investments

Cabo Verde - Hungary Bilateral Investment Treaty

Caribbean Forum States - United Kingdom Economic Partnership Agreement

Côte d'Ivoire – Portugal Bilateral Investment Treaty

European Union – Viet Nam Free Trade Agreement

Georgia – United Kingdom Strategic Partnership and Cooperation Agreement

India – Kyrgyzstan Bilateral Investment Treaty

New Zealand – Singapore Comprehensive and Enhanced Partnership Agreement

South African Customs Union – Mozambique – United Kingdom Economic Partnership Agreement

United Kingdom – Republic of Korea Free Trade Agreement

***2020***

Brazil – India Agreement on Cooperation and Facilitation of Investments

Chile – Ecuador Economic Complementation Agreement

European Union – United Kingdom Trade and Cooperation Agreement

Hong Kong Special Administrative Region, China – Mexico Bilateral Investment Treaty

Japan – United Kingdom Comprehensive and Enhanced Partnership Agreement

Republic of Moldova – United Kingdom Strategic Partnership, Trade and Cooperation Agreement

Ukraine – United Kingdom Free Trade and Strategic Partnership

***2021***

Australia – United Kingdom Free Trade Agreement

Chile – Paraguay Free Trade Agreement

China – European Union Comprehensive Agreement on Investment

Colombia – Spain Bilateral Investment Treaty

Democratic Republic of the Congo – Rwanda Bilateral Investment Treaty

European Union – Organisation of African, Caribbean and Pacific States Partnership Agreement

Iceland – Liechtenstein – Norway – United Kingdom Free Trade Agreement

Hungary – United Arab Emirates Bilateral Investment Treaty

***2022***

European Union – Angola Sustainable Investment Facilitation Agreement

Chile – European Union Interim Trade Agreement

Hungary – Oman Bilateral Investment Treaty

Indonesia – Switzerland Bilateral Investment Treaty

New Zealand – United Kingdom Free Trade Agreement

Pacific Alliance – Singapore Free Trade Agreement

Türkiye – Uruguay Bilateral Investment Treaty

***2023***

China – Ecuador Free Trade Agreement

European Free Trade Association – Republic of Moldova Free Trade Agreement

European Union – New Zealand Free Trade Agreement

**Fig. 1:**
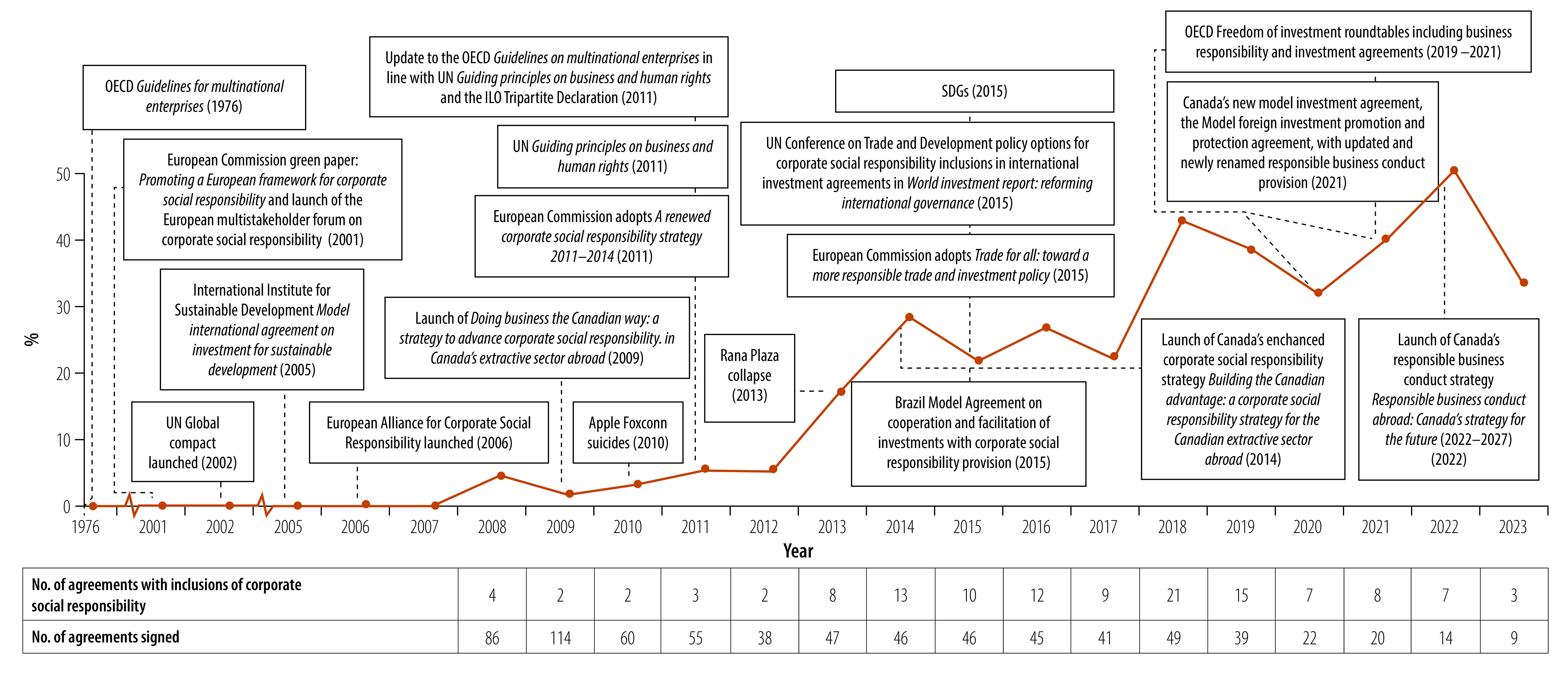
Percentage of international investment agreements signed each year with corporate social responsibility inclusions, in the context of corporate social responsibility development, 1976–2023

ILO: International Labour Organization; OECD: Organisation for Economic Co-operation and Development; SDGs: sustainable development goals; UN: United Nations.

The literature review identified significant international developments that have accompanied the rise of corporate social responsibility inclusions in international investment agreements (Fig. 1). The development of corporate social responsibility guidelines and principles such as the UN Guiding Principles on Business and Human Rights,^35^ UN Global Compact^7^ and the OECD *Guidelines for multinational enterprises on responsible business conduct*,^36^ have helped to formalize and legitimize corporate social responsibility through soft law.^6^ Tragedies involving investors have also elevated the importance of corporate social responsibility, allowing that businesses, particularly foreign investors operating in low- and middle-income countries, be held accountable for their impact on society.^2^^,^^37^ Examples of such tragedies were the Apple Foxconn suicides in 2010, which highlighted the poor working conditions at the electronics manufacturer contracted with Apple,^38^ and the Rana Plaza collapse in 2013, in which 1134 people died when a building owned and operated by a multinational firm collapsed.^39^ Furthermore, the emphasis of the SDGs and UN on the role of businesses in achieving the goals^40^ has further defined the socially acceptable behaviour of businesses.^8^

Data sources: Electronic Database of Investment Treaties and authors’ review of the literature^28^^,^^31^^–^^34^ as well as literature cited in the findings of the paper.

The parties including corporate social responsibility provisions in their international investment agreements represent a variety of regions and economies.^28^ Using WHO region categorizations, 23 of the agreements had a party from the African Region, 64 from the Region of the Americas, 11 from the South-East Asia Region, 65 from the European Region, 12 from the Eastern Mediterranean Region and 31 from the Western Pacific Region. The leading parties were Canada, which was a party to 21 of the 127 agreements, the European Union (EU) with 20, and Brazil with 16. Using World Bank gross national income classifications from October 2023,^41^ 103 of the agreements had a high-income party, 84 had an upper-middle-income party, 37 had a lower-middle-income party and 13 had a low-income party.

The inclusions were found both in the preambles and the provisions of the agreements, with 36 inclusions appearing in the preambles. In the provisions, there were 62 corporate social responsibility articles, eight responsible business conduct articles, and 31 combined responsible business conduct and corporate social responsibility articles. Furthermore, 60 inclusions of corporate social responsibility and/or responsible business conduct were embedded in other articles.

### Corporate social responsibility inclusions

We observed notable patterns and trends in the way corporate social responsibility inclusions in international investment agreements have been drafted. We identified six distinct categories within our typology, from minimal to high level of detail (Table 1). References to guidelines varied from general mentions through to the specific identification of guidelines, for example, the OECD *Guidelines for multinational enterprises on responsible business conduct*,^36^ that were developed in 1976, last updated in 2023 and are referenced in 43 international investment agreements reviewed. These guidelines cover topics ranging from human rights to the environment. The descriptions of corporate social responsibility in the agreements ranged from minimal (that is, simply a mention of the term) through to a high level of detail regarding both the definition and expectations regarding investor activity.

For instance, Article 14.17 of the Australia–Indonesia Comprehensive Economic Partnership Agreement (CEPA) (2018) is categorized as having a low level of detail in its corporate social responsibility inclusion because the article refers to standards, guidelines and principles of corporate social responsibility but does not name them or provide any description. On the other hand, Article 12.11 in the Free Trade Agreement Between the European Union and the Republic of Singapore (2018) is categorized as medium level. First, it refers to the specific corporate social responsibility principles, standards and guidelines such as the OECD *Guidelines for multinational enterprises on responsible business conduct*,^36^ the UN Global Compact^7^ and the International Labour Organization Tripartite Declaration.^42^ Second, it also provides a brief description of the corporate social responsibility activities through its contextual placement in an article on trade and investment promoting sustainable development, titled Trade and Sustainable Development.

**Fig. 2:**
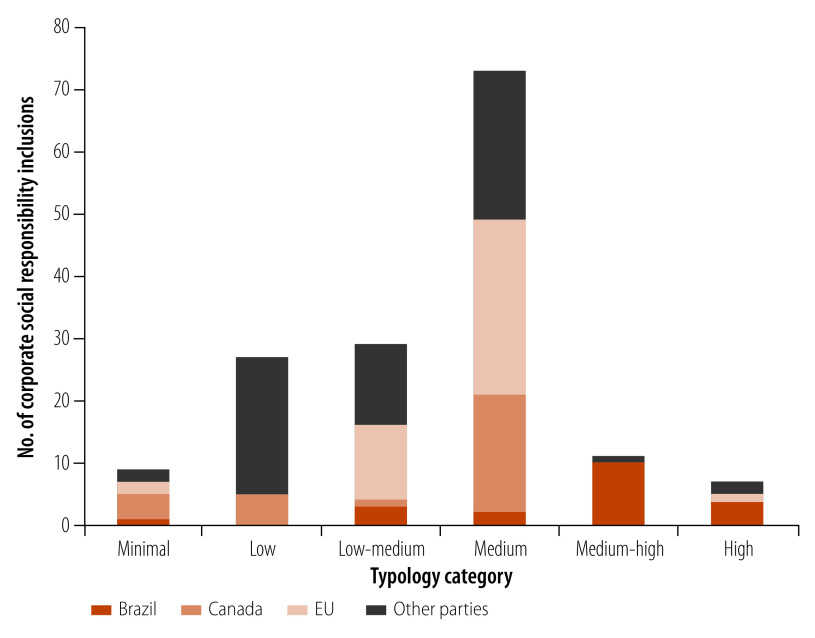
Corporate social responsibility inclusions by typology category and leading parties, 2023

Corporate social responsibility inclusions in international investment agreements have been led by three parties: Canada, the EU and Brazil (Fig. 2). Canada is a party to 21 agreements with corporate social responsibility inclusions, the highest of the parties in this data set. The majority are in the medium category (Fig.2). The EU is a party to 20 agreements and with most of the inclusions in the low-medium and medium categories (Fig. 2). A characteristic unique to these agreements is that 15 of them contain Trade and Sustainable Development chapters, or sections where corporate social responsibility provisions or references to corporate social responsibility are placed. Unique to the Brazilian corporate social responsibility provisions is their specification of investor expectations, rather than encouraging governments to motivate investors to abide by corporate social responsibility standards as in other agreements.

EU: European Union.

Note: Description of the criteria is available in Table 1.

### Health inclusions 

We found 36 explicit references to health alongside and within corporate social responsibility inclusions across 32 of the 127 (25%) agreements in the data set. Many of these inclusions integrate health protection and health policy explicitly into the description of the expected investor conduct and characteristics of corporate social responsibility. Twenty-nine references in corporate social responsibility provisions were outside of the preamble. Of particular note are the health-related corporate social responsibility clauses in 13 out of the 15 international investment agreements that Brazil is party to in the data set, which include provisions stating that industries operating in the host country as investors are not to seek exemptions to health policy. Using the typology, four of these provisions were categorized as high and nine as medium-high. For example, the Agreement on Cooperation and Facilitation of Investment Between the Federative Republic of Brazil and the United Mexican States (2015) includes a clause to specify that investors shall endeavour to:

“[R]efrain from seeking or accepting exemptions that are not established in the legal or regulatory framework relating to human rights, environment, health, security, work, tax system, financial incentives or other issues.”

The remaining seven references to health were in the preamble of their respective international investment agreements alongside corporate social responsibility inclusions. These inclusions provide a broad interpretive reference point for the agreement and serve to indicate broad policy commitment by the parties to protecting health. For example, in the Agreement Between the Government of Hungary and the Government of the Kyrgyz Republic for the Promotion and Reciprocal Protection of Investments (2020), the preamble includes the following clause:

“Seeking to ensure that investment is consistent with the protection of health, safety and the environment, the promotion and protection of internationally and domestically recognised human rights, labour rights, and internationally recognised standards of corporate social responsibility.”

## Discussion

Our analysis has identified a significant increase in corporate social responsibility inclusions in international investment agreements over the past 16 years, representing a shift towards balancing investor rights with investor obligations. The diversity of parties including corporate social responsibility in investment agreements suggests that efforts to balance these agreements are not constrained by geography or economy.

The corporate social responsibility provisions that were identified in this analysis are embedded in broader shifts within international investment agreements over the past 20 years. For example, the EU structure and placement of corporate social responsibility within Trade and Sustainable Development chapters provides framing and context for the interpretation of corporate social responsibility. The EU has stated that it views corporate social responsibility as a mechanism and set of values through which the SDGs will be implemented in its Member States as part of its holistic approach.^5^^,^^43^ In October 2015, the European Commission adopted a new trade and investment policy titled *Trade for all: towards a more responsible trade and investment policy*.^44^ The policy included corporate social responsibility provisions as part of an effort to make the EU’s trade and investment policy more responsible through three principles: effectiveness, transparency and values. Our findings reflect the European Commission’s development of a holistic and integrated approach over the last decade, particularly concerning corporate social responsibility, responsible business conduct, business and human rights and the SDGs. Similarly, the innovative and unique attributes of the corporate social responsibility provisions in agreements involving Brazil reflect its broader engagement with the international investment law regime.^45^ Historically, Brazil’s congress has not passed traditionally structured investment agreements due to a reluctance and resistance to the investor-State dispute arbitration, seeing an imbalance in favour of foreign investors in the arbitration mechanism.^45^^,^^46^ However, this limitation has not hindered foreign investment.^47^ Furthermore, the Brazilian government has recently developed its own bilateral investment treaty model, which has State-State arbitration rather than investor-State arbitration.^45^

The typology of corporate social responsibility inclusions in international investment agreements presented in this paper differentiates vague and low-detail corporate social responsibility inclusions from clearer and higher level of detail inclusions of corporate social responsibility. By developing a typology of corporate social responsibility inclusions, our analysis contributes to a better understanding of the nature and diversity of these provisions and highlights opportunities to strengthen the inclusion of specific investor expectations, including in relation to public health. Relatively limited explicit inclusion of health in corporate social responsibility is observed to date, and an opportunity exists to strengthen these inclusions as part of efforts towards increased accountability of corporate social responsibility related to public health. In particular, the provisions stating that industries operating in the host country as investors are not to seek exemptions to health policy are consistent with public health goals to reduce the influence of vested interests in health policy-making.^48^ These provisions draw attention to industry efforts to undermine the application of health policy, including through investor-State dispute mechanisms and other domestic avenues. By stating an explicit expectation that investors will not seek to limit or avoid health policy measures, these provisions also send a signal regarding the scope of investor rights and the priority placed on health policy.

The typology is purely descriptive and further research is needed to assess the strength or impact of these inclusions in the event of arbitration. There is a need for research into strong corporate social responsibility provisions, the development of responsible business conduct provisions and the potential benefits of both. Specifically, further legal research into these provisions would improve understanding of their legal implications in the event of arbitration and how they may impact on the occurrence of regulatory chill, which refers to governments’ reluctance to pursue new domestic health policy measures following a real or perceived threat of arbitration.^49^

International investment agreements can place positive obligations on foreign investors to act in alignment with government policy objectives through corporate social responsibility provisions. Corporate social responsibility inclusions form part of an effort to rebalance international investment agreements and expand their scope to include sustainable development and health. Understanding the nature of these provisions offers public health policy-makers a potential avenue to improve government oversight of corporate social responsibility, thereby increasing the accountability of powerful global corporations in relation to governments’ public health objectives.

## References

[1] Kercher K. Corporate social responsibility: impact of globalisation and international business. Enterp Gov EJournal. 2007;1(1). 10.53300/001c.6906

[2] Lahey T. Using bilateral investment treaties to promote corporate social responsibility and stimulate sustainable development. Rutgers Bus Law Rev. 2019. Available from: https://heinonline.org/HOL/LandingPage?handle=hein.journals/rutgblaj15&div=5&id=&page= [cited 2022 Jul 14].

[3] Friedman M. A Friedman doctrine- the social responsibility of business is to increase its profits. The New York Times. 1970 Sep 13. Available from: https://www.nytimes.com/1970/09/13/archives/a-friedman-doctrine-the-social-responsibility-of-business-is-to.html [cited 2022 Aug 5].

[4] Garriga E, Melé D. Corporate social responsibility theories: mapping the territory. J Bus Ethics. 2004 Aug 1;53(1/2):51–71. 10.1023/B:BUSI.0000039399.90587.34

[5] Corporate social responsibility, responsible business conduct, and business and human rights. Overview of progress. Brussels: European Commission; 2019. Available from: https://ec.europa.eu/docsroom/documents/34963 [cited 2022 Aug 5].

[6] Cîrlig RE. Business and human rights: from soft law to hard law? In: Sixth international conference on perspectives of business law on the third millennium; 2016 Nov 25–26; Bucharest, Romania. Bucharest: Bucharest University of Economic Studies; 2016. Available from: http://tribunajuridica.eu/arhiva/An6v22/16%20Cirlig.pdf [cited 2022 Jul 26].

[7] The ten principles of the United Nations Global Compact. New York: United Nations Global Compact. Available from: https://unglobalcompact.org/what-is-gc/mission/principles [cited 2023 Nov 25].

[8] Waleson J. Corporate social responsibility in EU comprehensive free trade agreements: towards sustainable trade and investment. Leg Issues Econ Integr. 2015;42(2):143–74. 10.54648/LEIE2015009

[9] Corporate social responsibility and responsibility. Brussels: European Commission; 2022. Available from: https://single-market-economy.ec.europa.eu/industry/sustainability/corporate-sustainability-and-responsibility_en [cited 2022 Aug 5].

[10] Tache CEP. Introduction to international investment law. Bucharest: Adjuris; 2020.

[11] Mann H. International investment agreements, business and human rights: key issues and opportunities. Winnipeg: International Institute for Sustainable Development; 2008. Available from: https://www.iisd.org/system/files/publications/iia_business_human_rights.pdf [cited 2022 Jul 14].

[12] Adeleke F. Human rights and international investment arbitration. S Afr J Hum Rights. 2016;32(1):48–70. 10.1080/02587203.2016.1162436

[13] Levashova Y. The role of corporate social responsibility in international investment law: the case of tobacco. In: Tench R, Sun W, Jones B, editors. Communicating corporate social responsibility: perspectives and practice (critical studies on corporate responsibility, governance and sustainability, Vol. 6). Bingley: Emerald Group Publishing Limited; 2014. pp. 131–53. 10.1108/S2043-9059(2014)0000006027

[14] Voon T, Mitchell AD. Philip Morris vs. tobacco control: two wins for public health, but uncertainty remains. In: Sauvant K, editor. Columbia FDI perspectives: perspectives on topical foreign direct investment issues. New York: Columbia Center on Sustainable Development; 2016.Available from: https://doi.org/, 10.7916/D87P8ZTG

[15] Gilmore AB, Fabbri A, Baum F, Bertscher A, Bondy K, Chang HJ, et al. Defining and conceptualising the commercial determinants of health. Lancet. 2023 Apr 8;401(10383):1194–213. 10.1016/S0140-6736(23)00013-2 36966782

[16] Garde A, Zrilič J. International investment law and non-communicable diseases prevention. J World Invest Trade. 2020 Oct 14;21(5):649–73. 10.1163/22119000-12340190

[17] Alschner W, Tuerk E. The role of international investment agreements in fostering sustainable development. In: Baetens F, editor. Investment law within international law: integrationist perspectives. Cambridge: Cambridge University Press; 2013. pp. 217–31. 10.1017/CBO9781139855921.014

[18] Sustainable development goal 3. New York: Department of Economic and Social Affairs; 2015. Available from: https://sdgs.un.org/goals/goal3 [cited 2023 Oct 31].

[19] Macassa G, Da J, Cruz F, McGrath C. Corporate social responsibility and population health. Health Sci J. 2017;11(5):1–6. 10.21767/1791-809X.1000528

[20] Ponte S, Richey LA, Baab M. Bono’s Product (RED) Initiative: corporate social responsibility that solves the problems of ‘distant others’. Third World Q. 2009 Mar 1;30(2):301–17. 10.1080/01436590802681074

[21] Babor TF, Robaina K. Public health, academic medicine, and the alcohol industry’s corporate social responsibility activities. Am J Public Health. 2013 Feb;103(2):206–14. 10.2105/AJPH.2012.300847 23237151 PMC3558773

[22] Herrick C. Shifting blame/selling health: corporate social responsibility in the age of obesity. Sociol Health Illn. 2009 Jan;31(1):51–65. 10.1111/j.1467-9566.2008.01121.x 18764803

[23] Herrick C. The post-2015 landscape: vested interests, corporate social responsibility and public health advocacy. Sociol Health Illn. 2016 Sep;38(7):1026–42. 10.1111/1467-9566.12424 27037612

[24] Monachino MS, Moreira P. Corporate social responsibility and the health promotion debate: an international review on the potential role of corporations. Int J Healthc Manag. 2014;7(1):53–9. 10.1179/2047971913Y.0000000058

[25] Yoon S, Lam TH. The illusion of righteousness: corporate social responsibility practices of the alcohol industry. BMC Public Health. 2013 Jul 3;13(1):630–630. 10.1186/1471-2458-13-630 23822724 PMC3706248

[26] Potvin Kent M, Pauzé E, Guo K, Kent A, Jean-Louis R. The physical activity and nutrition-related corporate social responsibility initiatives of food and beverage companies in Canada and implications for public health. BMC Public Health. 2020 Jun 9;20(1):890. 10.1186/s12889-020-09030-8 32517669 PMC7281932

[27] Mialon M, Crosbie E, Sacks G. Mapping of food industry strategies to influence public health policy, research and practice in South Africa. Int J Public Health. 2020 Sep;65(7):1027–36. 10.1007/s00038-020-01407-1 32728853

[28] Alschner W, Elsig M, Polanco R. Introducing the electronic database of investment treaties (edit): the genesis of a new database and its use. World Trade Rev. 2021 Feb;20(1):73–94. 10.1017/S147474562000035X

[29] World investment report 2015: reforming international investment governance. Geneva: United Nations Conference on Trade and Development; 2015. Available from: https://unctad.org/publication/world-investment-report-2015 [cited 2023 Feb 2].

[30] Mann H, von Moltke K, Peterson LE, Cosbey A. IISD model international agreement on investment for sustainable development. ICSID Rev - Foreign Invest Law J. 2005 Mar 1;20(1):91–145. 10.1093/icsidreview/20.1.91

[31] 2021 FIPA model – Summary of main changes. Ottawa: Government of Canada; 2021. Available from: https://www.international.gc.ca/trade-commerce/trade-agreements-accords-commerciaux/agr-acc/fipa-apie/2021_model_fipa_summary-2021_modele_apie_resume.aspx?lang=eng [cited 2022 Jul 14].

[32] OECD Guidelines for multinational enterprises: responsible business conduct matters. Paris: Organisation for Economic Co-operation and Development; 2022. Available from: https://mneguidelines.oecd.org/MNEguidelines_RBCmatters.pdf [cited 2022 Nov 30].

[33] Canada’s strategy for responsible business conduct abroad. Ottawa: Government of Canada; 2021. Available from: https://www.international.gc.ca/trade-commerce/rbc-cre/strategy-strategie.aspx?lang=eng [cited 2022 Aug 5].

[34] Freedom of investment roundtables: summary of discussions. Paris: Organisation for Economic Co-operation and Development; 2022. Available from: https://www.oecd.org/investment/investment-policy/oecdroundtablesonfreedomofinvestment.htm [cited 2022 Aug 5].

[35] Text of the UN Guiding Principles of Human Rights. London: Business and Human Rights Resource Centre; 2023. Available from: https://www.business-humanrights.org/en/big-issues/un-guiding-principles-on-business-human-rights/text-of-the-guiding-principles/ [cited 2023 Nov 25].

[36] OECD Guidelines for multinational enterprises on responsible business conduct. Paris: Organisation for Economic Co-operation and Development; 2023. Available from: https://www.oecd.org/publications/oecd-guidelines-for-multinational-enterprises-on-responsible-business-conduct-81f92357-en.htm [cited 2023 Nov 25].

[37] Gaukrodger D. Business responsibilities and investment treaties. Paris: Organisation for Economic Co-operation and Development; 2020. Available from: https://www.oecd.org/daf/inv/investment-policy/Consultation-Paper-on-business-responsibilities-and-investment-treaties.pdf [cited 2022 Aug 5].

[38] Cedillo Torres CA, Garcia-French M, Hordijk R, Nguyen K, Olup L. Four case studies on corporate social responsibility: do conflicts affect a company’s corporate social responsibility policy? Utrecht Law Rev. 2012 Nov;8(3):51–73. 10.18352/ulr.205

[39] Vijeyarasa R, Liu M. Fast fashion for 2030: using the pattern of the sustainable development goals (SDGs) to cut a more gender-just fashion sector. Cambridge: Cambridge University Press; 2022. Available from: https://www.cambridge.org/core/journals/business-and-human-rights-journal/article/fast-fashion-for-2030-using-the-pattern-of-the-sustainable-development-goals-sdgs-to-cut-a-more-genderjust-fashion-sector/326A2604C7FB89EAAC2B931B98F4C6A0 [cited 2023 Oct 31].

[40] The SDGs explained for business. United Nations Global Compact. New York: United Nations; 2023. Available from: https://unglobalcompact.org/sdgs/about [cited 2023 Nov 25].

[41] World Bank country and lending groups. Washington, DC: The World Bank; 2023. Available from: https://datahelpdesk.worldbank.org/knowledgebase/articles/906519-world-bank-country-and-lending-groups [cited 2023 Oct 31].

[42] Tripartite declaration of principles concerning multinational enterprises and social policy (MNE Declaration). Geneva: International Labour Organization; 2023 Available from: http://www.ilo.org/empent/Publications/WCMS_094386/lang--en/index.htm [cited 2023 Nov 25].

[43] A renewed EU strategy 2011–14 for corporate social responsibility. Brussels: European Commission; 2011. Available from: https://eur-lex.europa.eu/LexUriServ/LexUriServ.do?uri=COM:2011:0681:FIN:EN:PDF [cited 2022 Jul 14].

[44] Trade for all: Towards a more responsible trade and investment policy. Brussels: European Commission; 2014. Available from: https://op.europa.eu/en/publication-detail/-/publication/d90eda7c-7299-11e5-9317-01aa75ed71a1 [cited 2023 Nov 29].

[45] Monebhurrun N. Novelty in international investment law: the Brazilian agreement on cooperation and facilitation of investments as a different international investment agreement model. J Int Dispute Settl. 2017 Mar 1;8(1):79–100. 10.1093/jnlids/idv028

[46] History of the ICSID Convention. Washington, DC: World Bank; 1968. Available from: https://icsid.worldbank.org/sites/default/files/publications/History%20of%20the%20ICSID%20Convention/History%20of%20ICSID%20Convention%20-%20VOLUME%20II-2.pdf [cited 2022 Aug 8].

[47] Ofodile U. Africa-China bilateral investment treaties: a critique. Mich J Int Law. 2013 Jan 1;35(1):131–211.

[48] Global action plan for the prevention and control of NCDs 2013–2020. Geneva: World Health Organization; 2013. Available from: https://www.who.int/publications-detail-redirect/9789241506236 [cited 2023 Jun 5].

[49] Garton K, Swinburn B, Thow AM. The interface between international trade and investment agreements and food environment policymaking: a conceptual framework. Front Polit Sci. 2022 Dec 7;4:1–2. 10.3389/fpos.2022.996017

